# Respiratory Syncytial Virus Immunization Intention During Pregnancy and Infancy

**DOI:** 10.1001/jamanetworkopen.2026.25117

**Published:** 2026-07-24

**Authors:** Rachael M. Porter, Ilse Campos, Mohammed Shoaib, Tara M. Vogt, Walter Orenstein, Robert A. Bednarczyk, Lavanya Vasudevan

**Affiliations:** 1 Hubert Department of Global Health, Rollins School of Public Health, Emory University, Atlanta, Georgia; 2 National Center for Immunization and Respiratory Diseases, Centers for Disease Control and Prevention, Atlanta, Georgia; 3 Department of Medicine, Pediatrics, Epidemiology and Global Health, Emory University, Atlanta, Georgia

## Abstract

**Importance:**

Respiratory syncytial virus (RSV) infection is the leading cause of infant hospitalizations in the US. Two immunization options are available to protect infants against hospitalization—maternal vaccination during pregnancy or RSV antibody shot administration for infants after birth. Gaps in immunization coverage suggest missed opportunities to protect infants against RSV-associated hospitalization.

**Objective:**

To describe RSV immunization intentions, behaviors, and preferences among pregnant women and parents of young children (ages 0-5 years).

**Design, Setting, and Participants:**

For this survey study, 2 concurrent national cross-sectional surveys were implemented in April 2024. Participants were recruited from a representative online survey panel of US adults. Participants were 18 years or older and either pregnant or the parent of a young child. Data were analyzed from May to November 2025.

**Main Outcomes and Measures:**

The outcomes of interest were prevalence of positive intention to receive RSV antibody shot for child among pregnant women and parents and prevalence of positive intention to receive RSV maternal vaccine among pregnant women.

**Results:**

Of 1765 parents and 174 pregnant women, most were aged 30 years or older (parent: 78.1% [95% CI, 76.2%-80.0%]; pregnant women: 64.0% [95% CI, 56.8%-71.1%]). Among pregnant women eligible for vaccination, 55.5% (95% CI, 47.7%-63.3%) reported a positive intention toward maternal RSV vaccination. Among pregnant women with negative or unsure intention, 35.8% (95% CI, 24.3%-47.3%) would probably or definitely accept RSV vaccination following a strong health care practitioner recommendation. Among parents, 59.8% (95% CI, 57.5%-62.1%) reported a positive intention toward RSV antibody shot administration for their child. Among parents with negative or unsure intention, 27.0% (95% CI, 23.7%-30.2%) would probably or definitely accept the RSV antibody shot for their child following a strong health care practitioner recommendation. More participants preferred maternal vaccination (parents: 48.3% [95% CI, 46.0%-50.6%]; pregnant women: 58.2% (95% CI, 50.9%-65.6%]) over infant RSV antibody shot administration (parents: 9.4% (95% CI, 8.1%-10.8%]; pregnant women: 6.2% (95% CI, 2.6%-9.8%). Top reasons for negative or unsure intentions in both groups included lack of information and safety concerns.

**Conclusions and Relevance:**

In this survey study, pregnant women and parents of young children preferred maternal RSV vaccination for protecting infants against RSV infection. For some participants with negative or unsure intentions about RSV immunization, a strong health care practitioner recommendation shifted intention toward accepting immunization. Future research efforts should design and evaluate novel communication strategies for providing information about RSV immunization options to eligible families, in addition to strengthening health care practitioner recommendation in favor of immunization.

## Key Points

**Question:**

What are the self-reported intentions, behaviors, and preferences of pregnant women and parents of young children regarding maternal respiratory syncytial virus (RSV) vaccination during pregnancy or RSV antibody shot administration for infants after birth?

**Findings:**

In this survey study including 1765 parents and 174 pregnant women, parents and pregnant women reported a preference for maternal RSV vaccination compared with RSV antibody shot administration for infants. Lack of information and safety concerns were reported as key barriers to immunization uptake, and some participants with negative or unsure intention toward RSV immunization were more likely to accept RSV immunization if a health care practitioner strongly recommended it.

**Meaning:**

These findings suggest that a multipronged communication strategy, including via health care practitioner recommendations, is needed to communicate about RSV immunization options and their safety with families during pregnancy and infancy.

## Teaser

This survey study describes the intentions, behaviors, and preferences regarding maternal vaccination during pregnancy or respiratory syncytial virus antibody shot administration for infants after birth among pregnant women and parents of children younger than 5 years.

## Introduction

The leading cause of hospitalizations among children in the US is respiratory syncytial virus (RSV) infection.^1^ Among US children younger than 5 years, RSV infections are estimated to cause 58 000 to 80 000 hospitalizations and 100 to 300 deaths annually.^2,3^ The burden of RSV infections is greatest among infants younger than 6 months, with the highest age-specific hospitalizations occurring in infants at 1 month,^4^ emphasizing the need for RSV infection protection of infants as soon as possible after birth.^5^ RSV infections peak from October to April in most of the continental US, tracking with other seasonal respiratory viruses (eg, influenza), providing an opportunity to coordinate prevention efforts with other seasonal immunization campaigns.^6^

The optimal timing for infant RSV antibody shot administration is shortly before the RSV season begins (eg, October to November), or within a baby’s first week of life if born October through March, and ideally during the birth hospitalization. In 2023, 2 RSV-specific immunization products were approved in the US to protect infants: a maternal RSV vaccine administered at gestational age 32 to 36 weeks, with seasonal administration from September to January to protect infants via the passive transfer of antibodies in the womb,^7^ and infant immunization with a long-acting RSV monoclonal antibody (hereafter, *RSV antibody shot*) for infants aged younger than 8 months who are born during or entering into their first RSV season and whose mother did not receive the RSV vaccine during pregnancy.^8^ There is no preferential recommendation between products, and families can select either product depending on eligibility and availability.^9^ In June 2025, an additional monoclonal antibody product was recommended for use in infants younger than 8 months whose mothers were not vaccinated during pregnancy, expanding the options for RSV immunization.^10,11,12^

In clinical studies, both maternal RSV vaccination and antibody shot administration have been shown to reduce RSV-associated hospitalizations among infants, reinforcing the impact of immunization as a prevention strategy.^13,14^ Despite this evidence of effectiveness, RSV immunization coverage across 33 states and the District of Columbia from the first full season of availability (2023-2024) was only 29% for infants who were protected through either maternal vaccination (10%) or RSV antibody shot administration (19%).^15^ The subsequent 2024 to 2025 season showed some improvements in coverage, with the proportion of infants protected by either maternal vaccination or the RSV antibody shot administration estimated to increase to 66% in February 2025.^13^ However, the gaps in coverage reflect missed opportunities for preventing RSV-related hospitalization among infants. A better understanding of factors contributing to the gaps is necessary to design strategies that support families in their RSV immunization decisions. For that purpose, we conducted 2 parallel, national, online cross-sectional surveys in April 2024 among pregnant women and among parents of young children (ages 0-5 years) to assess their RSV immunization intention, behaviors, and preferences. Study findings may inform strategies to improve support for RSV immunization decisions for infants.

## Methods

This survey study was approved by the Emory University institutional review board, meeting the criteria for exemption from further review and approval under 45 CFR 46.104(d)(2)(i). Staff from the Centers for Disease Control and Prevention (CDC) only had access to aggregated data, and it was determined the CDC was not engaged in human participants research, thus approval from CDC’s institutional review board was not required. Panel participants who clicked the link and completed the forms to proceed engaged in an implied consent. This study is reported in accordance with the American Association for Public Opinion Research (AAPOR) reporting guideline.

### Study Design

Two concurrent national cross-sectional surveys were conducted in April 2024 among pregnant women and parents of young children (ages 0-5 years) at the time of the survey. The survey asked about intentions and behaviors related to all vaccines recommended for children from birth to age 18 months, as well as the RSV immunization products.^9^ Survey recruitment has been previously reported.^16^ Briefly, survey participants were recruited from a nationally representative panel of US adults with a sampling frame covering nearly 100% of US households.^17^ Panel members received a survey link from the panel administrator, and those endorsing the implied consent script were redirected to self-administer the survey using an online survey platform (hosted on Qualtrics). Due to the estimated smaller pool of available pregnant panel members, participants who were both a parent and pregnant at the time of the survey were routed to the pregnancy survey. Eligible participants were 18 years or older, spoke English primarily, and self-reported pregnancy and/or were parents of a child aged 0 to 5 years. We retain full ownership of the data. All questions used for this analysis were voluntary (eAppendix in Supplement 1). Some questions were adapted from a previous survey.^18^

The survey included questions about infant RSV immunization preferences, RSV immunization intention or behaviors for the child, and maternal RSV vaccination intention or behaviors during pregnancy. Demographic characteristics were shared by the panel provider (ie, not collected as part of the survey), and have been previously reported.^16^ The potential for desirability bias was mitigated via self-administration of the survey by participants and by not collecting personally identifiable information associated with survey responses. The potential for selection bias was mitigated through the recruitment of survey participants from a nationally representative panel of US adults.

### Statistical Analysis

Descriptive analyses were conducted using SAS statistical software version 9.5 (SAS Institute Inc), with applied geodemographically calibrated sample weights provided by the survey panel company. Categorical variables were described with frequencies and percentages. Proportions between groups were compared descriptively due to small numbers in some response categories. No inferential statistical tests were performed due to the descriptive nature of the study. Missing data were excluded, and the numbers of missing responses per question are reported. Weighted percentages and accompanying 95% CIs are reported for ease of interpretation. Diverging stacked bar charts were used to visualize questions with responses on a Likert scale, with all related scale questions aligned on a central value and categorically neutral responses represented as nonpositive responses. Data were analyzed from May to November 2025.

## Results

A sample of 9855 panel members received the survey invitation. Of those, 6189 (excluding breakoffs) responded to the invitation, and 1939 qualified for the survey (inclusive of those who were either pregnant or a parent), yielding a final stage completion rate of 62.8% and a qualification rate of 31.3%. Data from 1765 parents of young children and 174 pregnant women were included in the analysis.

Demographic characteristics of the participants have been previously published.^16^ In brief, most participants were aged at least 30 years (pregnant women: 64.0% [95% CI, 56.8%-71.1%]; parents: 78.1% [95% CI, 76.2%-80.0%]), and resided in an urban setting (pregnant women: 81.0% [95% CI, 75.0%-86.9%]; parents: 82.4% [95% CI, 80.6%-84.2%]). Among pregnant women, 62.4% (95% CI, 55.2%-69.6%) reported a prior birth. Most participants had heard of RSV infection before taking the survey (pregnant women: 83.8% [95% CI, 78.3%-89.2%]; parents: 89.3% [95% CI, 87.9%-90.7%]).

**Figure 1.**
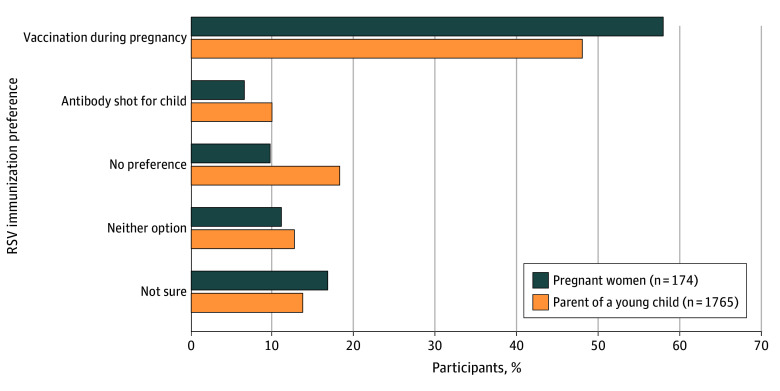
Bar Graph of Preferences for Respiratory Syncytial Virus (RSV) Immunization for Child, Among Pregnant Women or a Parent of a Young Child

**Figure 2.**
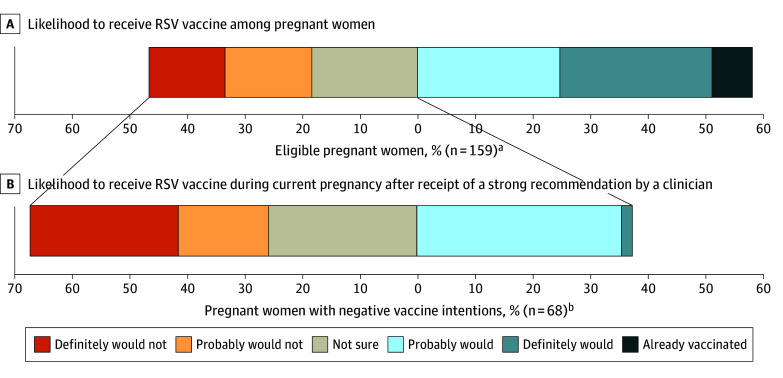
Component Bar Graph of Respiratory Syncytial Virus (RSV) Vaccination Intention Among Pregnant Women ^a^15 Pregnant women excluded (14 at >36 weeks’ gestation and not eligible to receive RSV vaccine this pregnancy; 1 did not answer). ^b^Pregnant women who originally answered they definitely would not, probably would not, or were not sure about receipt of an RSV vaccine in pregnancy.

All participants were asked about their preference between the 2 RSV immunization options (Figure 1). More participants reported a preference for maternal vaccination (pregnant women: 58.2% [95% CI, 50.9%-65.6%]); parents: 48.3% [95% CI, 46.0%-50.6%]) over the RSV antibody shot administration for their child (pregnant women: 6.2% [95% CI, 2.6%-9.8%]; parents: 9.4% [95% CI, 8.1%-10.8%]). Pregnant women were asked about their likelihood of receiving the RSV vaccine during their current pregnancy, and 10.3% (95% CI, 5.8%-14.8%) of the full sample of 174 pregnant women were ineligible, as they had more than 36 weeks’ gestation (ie, beyond the recommended window for vaccination during pregnancy) (Figure 2). Among the remaining pregnant women, 55.5% (95% CI, 47.7%-63.3%) reported positive intentions and 44.5% (95% CI, 36.7%-52.3%) reported negative or unsure intentions to get the RSV vaccine during pregnancy. Among pregnant women who reported negative or unsure intention, top reasons included not knowing enough about RSV or the vaccine to make a decision (50.4% [95% CI, 38.6%-62.1%]), worry about the safety of the vaccine for their child (38.6% [95% CI, 27.1%-50.0%]), and worry about the safety of vaccine for themselves (33.0% [95% CI, 21.9%-44.1%]) (eTable 1 in Supplement 1).

Among pregnant women with a negative or unsure intention about getting the RSV vaccine, 35.8% (95% CI, 24.3%-47.3%) said they would definitely or probably receive the RSV vaccine during pregnancy if a health care practitioner strongly recommended it. Among pregnant women who would shift to a positive intention after a strong health care practitioner recommendation, 9.4% (95% CI, 2.1%-16.8%) originally were not sure, 20.0% (95% CI, 6.1%-34.0%) probably would not, and 6.3% (95% CI, 0.4%-12.2%) definitely would not get the maternal RSV vaccine. Among pregnant women with negative or unsure intention, 64.2% (95% CI, 52.7%-75.8%) would not accept the vaccine during pregnancy even if a health care practitioner strongly recommended it.

**Figure 3.**
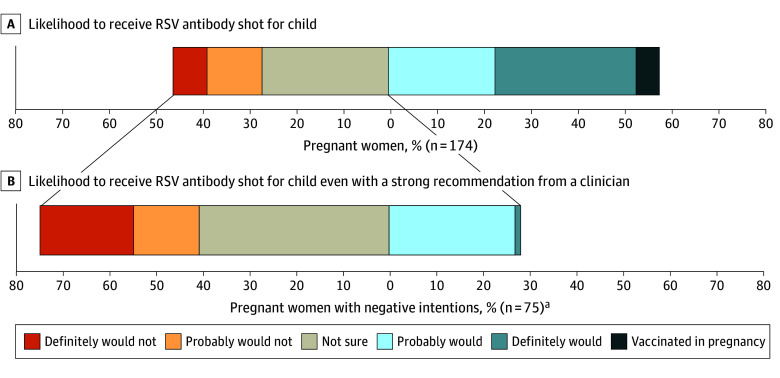
Component Bar Graph of Respiratory Syncytial Virus (RSV) Antibody Shot Intention for Child Among Pregnant Women ^a^Pregnant women who originally answered they definitely would not, probably would not, or were not sure about receipt of an RSV antibody shot for their child.

Pregnant women were asked about their intention to immunize their child, if eligible, with the RSV antibody shot, and 55.7% (95% CI, 48.3%-63.1%) reported a positive intention (Figure 3). Among pregnant women reporting negative or unsure intention, top reasons included not knowing enough about RSV or the RSV antibody shot to make a decision (53.9% [95% CI, 42.8%-65.0%]), worry about the long-term safety of the RSV antibody shot for the child (41.4% [95% CI, 30.4%-52.4%]), and worry about short-term safety of the RSV antibody shot for the child (23.8% [95% CI, 14.3%-33.3%]) (eTable 2 in Supplement 1).

Among pregnant women with negative or unsure intentions about immunizing their child, 27.4% (95% CI, 17.2%-37.6%) would change their mind if a health care practitioner strongly recommended the RSV antibody shot for their child. Among pregnant women who would shift to positive intention after a strong health care practitioner recommendation, 26.7% (95% CI, 13.3%-40.0%) originally were not sure and 0.7% (95% CI, 0.0%-2.1%) originally said they definitely would not get the RSV antibody shot for their child. Among pregnant women with negative or unsure intention, 72.6% (95% CI, 62.4%-82.9%) would not accept the RSV antibody shot for their child even if a health care practitioner strongly recommended it.

**Figure 4.**
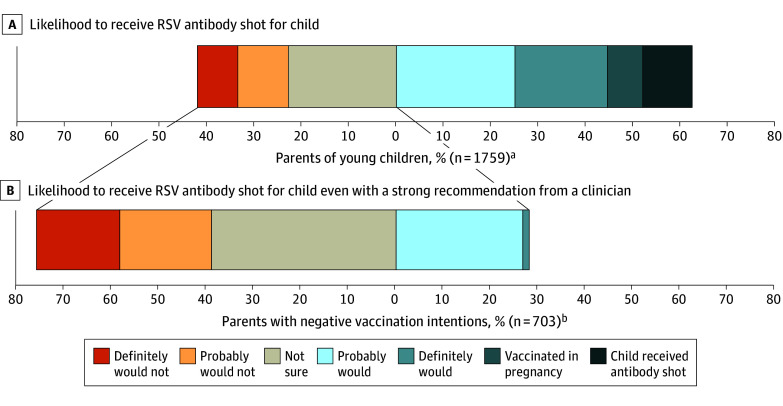
Component Bar Graph of Respiratory Syncytial Virus (RSV) Antibody Shot Intention for Child Among Parents of Young Children ^a^Six Parents did not answer and were excluded. ^b^Parents who originally answered they definitely would not, probably would not, or were not sure about receipt of an RSV antibody shot for their child.

When parents of young children were asked about their intention to immunize their child, if eligible, with the RSV antibody shot, 59.8% (95% CI, 57.5%-62.1%) reported a positive intention, and among those, 10.0% (95% CI, 8.6%-11.4%) had already immunized their child with the RSV antibody shot (Figure 4). Among parents who reported a negative or unsure intention to immunize their child, top reasons included worry about the long-term safety of the RSV antibody shot for the child (40.0% [95% CI, 36.4%-43.6%]), not knowing enough about RSV or the RSV antibody shot to make a decision (38.6% [95% CI, 35.1%-42.2%]), and worry about short-term safety of the RSV antibody shot for the child (27.6% [95% CI, 24.3%-30.9%]) (eTable 2 in Supplement 1). Among parents with a negative or unsure intention, 27.0% (95% CI, 23.7%-30.2%) would change their mind if a health care practitioner strongly recommended the RSV antibody shot for their child, including 25.7% (95% CI, 22.3%-28.7%) who said they probably would and 1.3% (95% CI, 0.4%-2.1%) who said they definitely would. Among parents who shifted to a positive intention after a strong health care practitioner recommendation, 21.6% (95% CI, 17.3%-25.9%) originally were not sure, 4.0% (95% CI, 1.8%-6.2%) originally probably would not, and 1.4% (95% CI, 0.0%-3.0%) originally definitely would not get the RSV antibody shot for their child. Among parents with a negative or unsure intention, 73.0% (95% CI, 69.8%-76.4%) would not accept the RSV antibody shot for their child, even if a health care practitioner strongly recommended it.

**Figure 5.**
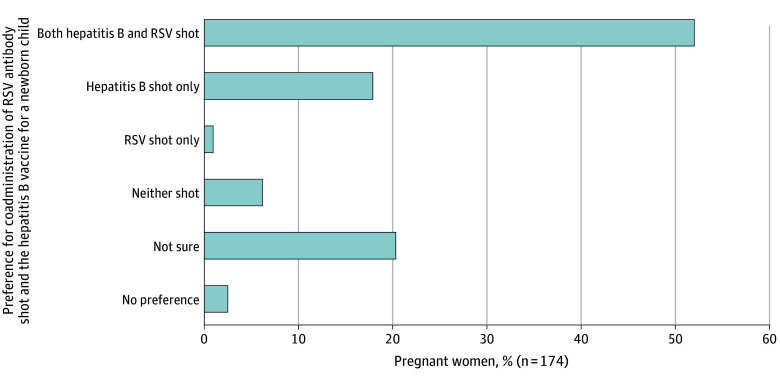
Bar Graph of Preferences Among Pregnant Women for Coadministration of Respiratory Syncytial Virus (RSV) Antibody Shot and the Hepatitis B Vaccine for Newborn Child

All pregnant women, regardless of RSV immunization intentions, were asked about the preferred location for their child to receive the RSV antibody shot (eFigure in Supplement 1). The most common responses included hospital or birthing center (34.2% [95% CI, 27.2%-41.3%]), followed by an outpatient pediatrician’s office (21.9% [95% CI, 15.7%-28.0%]). Pregnant women were asked about their preference for coadministration of the RSV antibody shot with the hepatitis B vaccine to the infant after birth if a health care practitioner recommended coadministration (Figure 5). More participants preferred coadministration (52.5% [95% CI, 45.0%-59.9%]), followed by 20.3% (95% CI, 14.3%-26.3%) of parents reporting they were unsure, and 17.8% (95% CI, 12.1%-23.5%) of parents preferring to get the hepatitis B shot alone. Notably, 6.0% (95% CI, 2.5%-9.6%) reported they preferred neither shot to be administered at the hospital or birthing center, even if a health care practitioner recommended coadministration.

## Discussion

The findings of this survey study on RSV immunization suggest that parents and pregnant women prefer vaccination during pregnancy, compared with RSV antibody shot administration during infancy, to protect their child against RSV infection. Our findings also suggest that individuals with negative or unsure intentions lacked the information they needed to feel confident in making RSV immunization decisions. While a health care practitioner recommendation may sway some participants to accept immunization, alternative strategies may be necessary to reach others.

Our first major finding is that a strong health care practitioner recommendation for RSV immunization shifted negative or unsure intentions of some participants. Health care practitioner recommendation has been shown previously to be associated with vaccination during pregnancy.^19^ Our findings suggest that there is a continued need for increasing opportunities for effective patient–health care practitioner communication around RSV immunization options. This finding is particularly salient in light of our prior work that found high uncertainty around vaccination decision-making, particularly during pregnancy.^16^ For individuals who prefer other information sources while making immunization decisions, future research could examine communication strategies and channels, including web-based, social media, or other online opportunities (eg, podcasts) as a means to reach families outside of the health care setting. Health care practitioner recommendation or other information sources may not sway the small group of pregnant women and parents who firmly state they do not want RSV immunization for themselves or their infants.

The second major finding of our study is that a top reason for refusal of both the RSV vaccine in pregnancy or RSV antibody shot for infants was lack of information about RSV, the RSV antibody shot, and the RSV vaccine in pregnancy, despite the finding that most participants had heard of RSV infection prior to the survey. This highlights the need for additional educational strategies to improve knowledge and awareness around the specifics of RSV prevention products, including their safety and effectiveness at preventing RSV-related hospitalizations. Additionally, among pregnant women in particular, concerns about safety for self and child (both long and short term) were cited as top concerns after lack of information as reasons for antibody shot and vaccine refusal. Our focus on RSV prevention products adds to a robust literature that explores the drivers of immunization decisions among pregnant women, which have primarily focused on other recommended vaccines during pregnancy (influenza, Tdap, and COVID-19). These results are in alignment with existing literature on the association between immunization and safety concerns among pregnant women, including safety concerns as top drivers for immunization decisions among pregnant women.^20,21,22^

The surveys were fielded in April 2024, which was at the end of the first season in which RSV prevention products became available and were recommended for use. A 2025 analysis of the 2023 to 2024 coverage estimates highlights several factors that might have contributed to low RSV immunization coverage, including limited supply and availability of products at preferred immunization locations, the complexity and ambiguity of recommendations between maternal RSV vaccination and RSV antibody shots, and finally, cost, as private health insurers have a 1-year grace period after Advisory Committee on Immunization Practices recommends an immunization product to include it in full health coverage, as outlined by the Patient Protection and Affordable Care Act.^15^ As these products are relatively new, issues of availability, accessibility, acceptability, and knowledge among the eligible community will continue to impact uptake, as seen through varying coverage estimates reported. Therefore, it may be important to reexamine reasons for refusal in future work to understand evolving immunization intentions and reasons for refusal of RSV vaccination and RSV antibody shot administration.

Finally, our findings on intentions for RSV immunization (55.5% of pregnant women with ≤36 weeks’ gestation had positive intention to receive or had received an RSV vaccine in pregnancy; 55.7%-59.8% of pregnant women and parents had positive intention to receive or had received an RSV antibody shot for their infants) are lower than estimates published by Gidengil et al^18^ in 2023 (61% willingness to receive RSV vaccine in pregnancy, 70% willingness for infant to receive RSV antibody shot). We hypothesize that the differences in estimates between our surveys and other surveillance reports relate to timing of survey administration. Reported estimates from Gidengil et al^18^ were from the season preceding RSV immunization product availability (2022-2023), while the surveys for this analysis were conducted at the tail end of the first RSV season with immunization product availability (2023-2024).

### Strengths and Limitations

This study has some strengths, including the national scope of the surveys and the use of survey weights for increasing the representativeness of the findings to the underlying population. In addition, the survey self-administration and national recruitment strategies potentially minimize desirability and selection bias, respectively.

This study also has some limitations, including the small sample of pregnant women included, contributing to small counts in some response options and inability to provide stratified results across different demographic characteristics. A second limitation is the independent nature of the 2 surveyed groups, precluding our ability to comment definitively on the associations between vaccination intentions in pregnancy and vaccination decisions among parents. Third, stated intentions may differ from actual vaccination behavior (eg, due to logistical challenges in getting vaccinated), and reports of any maternal or infant RSV immunization were not confirmed.

## Conclusions

In this survey study of pregnant women and parents of young children, participants preferred RSV vaccination during pregnancy as the primary means to protect against RSV infection in infancy. The survey findings highlight an information gap as the key barrier to immunization and reinforce the importance of health care practitioner recommendations to encourage immunization. Interventions delivered during pregnancy and early parenthood are important and timely to build knowledge about infant care and well-being, including immunization strategies to protect infants from RSV infection. Longitudinal studies from pregnancy through early parenthood may shed light on the relationships among immunization intentions, preferences, and behaviors and how they evolve as families interact with the health care system. Future studies may also ascertain the effectiveness of strategies for supporting infant RSV immunization decisions, including communication strategies implemented outside of traditional patient–health care practitioner interactions. Finally, future work should continue to explore strategies for strengthening health care practitioner recommendations for immunization during pregnancy and for young children.

## Notes

Supplement 1. eFigure. Preferences for location of RSV antibody shot administration for infants among pregnant women eAppendix. Excerpt of questions used to assess respiratory syncytial virus immunization intentions, behaviors, and preferences during pregnancy and infancy in 2 national surveys in the US, April 2024 eTable 1. Distribution of reasons for negative/unsure intention to receive respiratory syncytial virus vaccine during pregnancy, among pregnant women eTable 2. Distribution of reasons for negative/unsure intention to accept respiratory syncytial virus antibody shot for child, among pregnant women and parents

Supplement 2. Data Sharing Statement
