# Supplementary material for: Respiratory Syncytial Virus Immunization Intention During Pregnancy and Infancy
Source: JAMA Netw Open. 2026 Jul 24;9(7):e2625117. doi: 10.1001/jamanetworkopen.2026.25117 (PMC13401208; doi:10.1001/jamanetworkopen.2026.25117)
Supplement: Supplement 1. — eFigure. Preferences for location of RSV antibody shot administration for infants among pregnant women eAppendix. Excerpt of questions used to assess respiratory syncytial virus immunization intentions, behaviors, and preferences during pregnancy and infancy in 2 national surveys in the US, April 2024 eTable 1. Distribution of reasons for negative/unsure intention to receive respiratory syncytial virus vaccine during pregnancy, among pregnant women eTable 2. Distribution of reasons for negative/unsure intention to accept respiratory syncytial virus antibody shot for child, among pregnant women and parents [file jamanetwopen-e2625117-s001.pdf]

Supplemental Online Content

Porter RM, Campos I, Shoaib M, et al. Respiratory syncytial virus immunization intention during pregnancy and infancy. *JAMA Netw Open*. 2026;9(7):e2625117.
doi:10.1001/jamanetworkopen.2026.25117

eFigure. Preferences for location of RSV antibody shot administration for infants among pregnant women
eAppendix. Excerpt of questions used to assess respiratory syncytial virus immunization intentions, behaviors, and preferences during pregnancy and infancy in 2 national surveys in the US, April 2024
eTable 1. Distribution of reasons for negative/unsure intention to receive respiratory syncytial virus vaccine during pregnancy, among pregnant women
eTable 2. Distribution of reasons for negative/unsure intention to accept respiratory syncytial virus antibody shot for child, among pregnant women and parents

This supplemental material has been provided by the authors to give readers additional information about their work.

© 2026 Porter RM et al. *JAMA Network Open*.

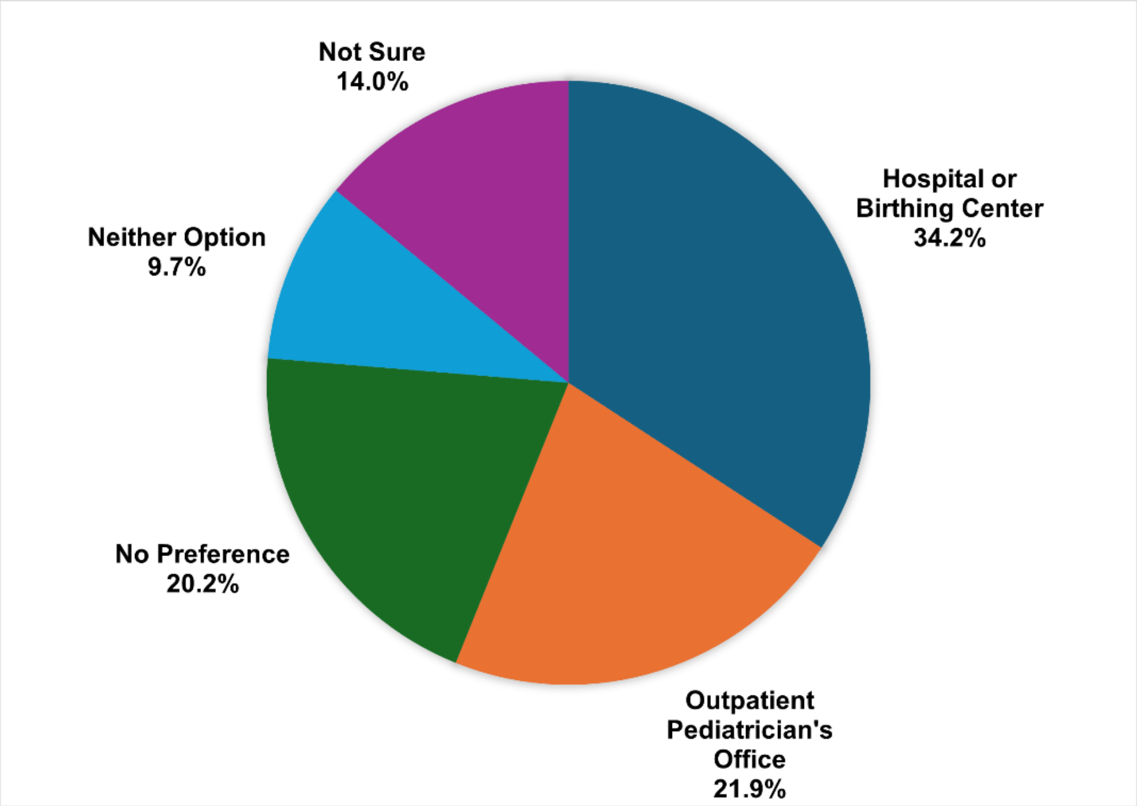

eFigure 1: Preferences for location of RSV antibody shot administration for infants among pregnant women (n=174).

© 2026 Porter RM et al. *JAMA Network Open*.

eFile 1: Excerpt of questions used to assess Respiratory syncytial virus immunization intentions, behaviors, and preferences during pregnancy and infancy in two national surveys in the United States, April 2024.

Note: Survey questions were adapted from an instrument developed by Scherer, Gidengil, and colleagues.[19]

SURVEY 1: FOR PARENTS

Start of Block: RSV vaccination intention

Statement: The following questions ask about RSV or Respiratory Syncytial Virus.

Q18 Have you heard of RSV or Respiratory Syncytial Virus before?

- ○ Yes (1)
- ○ No (0)

RSV statement Respiratory syncytial (sin-SISH-uhl) virus, or RSV, is a common respiratory virus that usually causes mild, cold-like symptoms. Most people recover in a week or two, but RSV can be serious, especially for infants and older adults. RSV is the most common cause of bronchiolitis (inflammation of the small airways in the lung), pneumonia (infection of the lungs), and hospitalization in children younger than 1 year of age in the United States.

End of Block: RSV vaccination intention

Start of Block: RSV vaccination for child

Statement: The following questions ask about RSV antibody shots for your youngest child.

Q19 Antibodies are proteins that are made by your body when it fights against an infection. Antibodies help prevent the infection from attacking the body. Children who are too young to make antibodies against RSV themselves may be able to get RSV antibodies (e.g., as a shot) to protect them from RSV infection. Children can also receive RSV antibodies from their mother, if the mother is vaccinated against RSV during pregnancy. An FDA-approved, CDC-recommended RSV antibody shot called nirsevimab (brand name: Beyfortus) is available and recommended for children younger than 8 months of age during RSV season (fall/winter/spring).

If your child was eligible, how likely would you be to have your youngest child get the antibody shot?

- ◯ Definitely would not have my child get the antibody shot (1)
- ◯ Probably would not have my child get the antibody shot (2)
- ◯ Not sure about having my child get the antibody shot (3)
- ◯ Probably would have my child get the antibody shot (4)
- ◯ Definitely would have my child get the antibody shot (5)
- ◯ Already got the antibody shot for my child (6)
- ◯ Already received RSV vaccination during pregnancy (7)

Display This Question:

If Q19 = 1

Or Q19 = 2

Or Q19 = 3

Q20 Which of the following, if any, are reasons that you "${Q19/ChoiceGroup/SelectedChoices}"? **Please select all that apply.**

- ▢ My child would not be at risk of getting RSV (1)
- ▢ My child would not get very sick if they got RSV (2)
- ▢ Worry about the short-term safety of an RSV antibody shot for my child (3)
- ▢ Worry about the long-term safety of an RSV antibody shot for my child (4)
- ▢ Worry that an RSV antibody shot might cause RSV (5)
- ▢ Worry that an RSV antibody shot might make future RSV infection worse (6)
- ▢ Worry about the cost of an RSV antibody shot (7)

© 2026 Porter RM et al. *JAMA Network Open*.

- [ ] I’ve had RSV infection during this pregnancy (8)
- [ ] An RSV antibody shot would not work very well at preventing RSV for my child (9)
- [ ] Putting existing antibodies into someone is against my religious beliefs (10)
- [ ] I do not like needles (11)
- [ ] I do not think RSV is real (12)
- [ ] I wouldn’t trust an RSV antibody shot (13)
- [ ] I wouldn’t have time to have my child get the antibody shot (14)
- [ ] I feel like I don’t know enough about RSV or the antibody shot to make a decision (15)
- [ ] My child’s healthcare provider recommended against the antibody shot (16)
- [ ] Other - please specify: (17) ____

Display This Question:

If Q19 = 1

Or Q19 = 2

Or Q19 = 3

© 2026 Porter RM et al. *JAMA Network Open*.

Q21 If a healthcare provider strongly recommended your child get an RSV antibody shot and your child was eligible for the antibody shot, how likely would you be to have your child get the antibody shot?

- ◯ Definitely would not have my child get the antibody shot (1)
- ◯ Probably would not have my child get the antibody shot (2)
- ◯ Not sure about having my child get the antibody shot (3)
- ◯ Probably would have my child get the antibody shot (4)
- ◯ Definitely would have my child get the antibody shot (5)

Q22 If you could protect your child by getting the RSV vaccine during pregnancy or by giving your child an RSV antibody shot, which option would you choose?

- ◯ Vaccination during pregnancy (1)
- ◯ Antibody shot for child (2)
- ◯ No preference (3)
- ◯ Neither option (4)
- ◯ Not sure (5)

© 2026 Porter RM et al. *JAMA Network Open*.

SURVEY 2: FOR PREGNANT WOMEN

Start of Block: RSV vaccination during pregnancy

Statement: The following questions ask about RSV or Respiratory Syncytial Virus.

Q18 Have you heard of RSV or Respiratory Syncytial Virus before?

- ○ Yes (1)
- ○ No (0)

Statement: Respiratory syncytial (sin-SISH-uhl) virus, or RSV, is a common respiratory virus that usually causes mild, cold-like symptoms. Most people recover in a week or two, but RSV can be serious, especially for infants and older adults. RSV is the most common cause of bronchiolitis (inflammation of the small airways in the lung), pneumonia (infection of the lungs), and hospitalization in children younger than 1 year of age in the United States.

Statement: The following questions ask about RSV vaccination during your current pregnancy.

Q19 An FDA-approved, CDC-recommended RSV vaccine is available and recommended for pregnant people during 32-36 weeks of pregnancy from September through January. Vaccination in pregnancy protects children from RSV infection after birth. How likely would you be to get the RSV vaccine during this current pregnancy to protect your child from RSV infection after birth?

© 2026 Porter RM et al. *JAMA Network Open*.

- ◯ Definitely would not get the vaccine during this pregnancy (1)
- ◯ Probably would not get the vaccine during this pregnancy (2)
- ◯ Not sure about getting the vaccine during this pregnancy (3)
- ◯ Probably would get the vaccine during this pregnancy (4)
- ◯ Definitely would get the vaccine during this pregnancy (5)
- ◯ Already got the vaccine during this pregnancy (6)

© 2026 Porter RM et al. *JAMA Network Open*.

Display This Choice:

If Q1 = 3

○ I am >36 weeks pregnant so I am not eligible (7)

Display This Question:

If Q19 = 1

Or Q19 = 2

Or Q19 = 3

Q20 Which of the following, if any, are reasons that you ${Q19/ChoiceGroup/SelectedChoices}? **Please select all that apply:**

▢ My child would not be at risk of getting RSV after being born (1)

▢ My child would not get very sick if they got RSV after being born (2)

▢ Worry about the safety of an RSV vaccine for myself (3)

▢ Worry about the safety of an RSV vaccine for my child (4)

▢ Worry that an RSV vaccine might cause RSV (5)

▢ Worry that an RSV vaccine might make future RSV infection worse (6)

▢ Worry about the cost of an RSV vaccine (7)

▢ I've already had RSV (8)

▢ An RSV vaccine during pregnancy would not work very well at preventing RSV for my child after they are born (9)

▢ I am planning to get the RSV antibody shot for my child after they are born (10)

▢ My healthcare provider recommended against the RSV vaccine (11)

▢ Vaccines are against my religious beliefs (12)

© 2026 Porter RM et al. *JAMA Network Open*.

☐ I do not like needles (13)

☐ I do not think RSV is real (14)

☐ I wouldn't trust an RSV vaccine (15)

☐ I wouldn't have time to get vaccinated (16)

☐ I have trouble finding a place that offers the RSV vaccine (17)

☐ I feel like I don't know enough about RSV or the vaccine to make a decision (18)

☐ Other - please specify: (19) ____

Display This Question:

If Q19 = 1

Or Q19 = 2

Or Q19 = 3

Q21 If your healthcare provider strongly recommended you get an RSV vaccine during pregnancy, and you were eligible for the vaccine, how likely would you be to get the vaccine?

○ Definitely would not get the vaccine during pregnancy (1)

○ Probably would not get the vaccine during pregnancy (2)

○ Not sure about getting the vaccine during pregnancy (3)

○ Probably would get the vaccine during pregnancy (4)

○ Definitely would get the vaccine during pregnancy (5)

© 2026 Porter RM et al. *JAMA Network Open*.

End of Block: RSV vaccination during pregnancy

Start of Block: RSV vaccination for child

Statement: The following questions ask about RSV antibody shots for your child, once your child is born.

Q22 Antibodies are proteins that are made by your body when it fights against an infection. Antibodies help prevent the infection from attacking the body. Children who are too young to make antibodies against RSV themselves may be able to get RSV antibodies (e.g., as a shot) to protect them from RSV infection. Children can also receive RSV antibodies from their mother, if the mother is vaccinated against RSV during pregnancy. An FDA-approved, CDC-recommended RSV antibody shot called nirsevimab (brand name: Beyfortus) is available and recommended for children younger than 8 months of age during RSV season (fall/winter/spring). How likely would you be to have your child get the antibody shot, once eligible?

- ◯ Definitely would not have my child get the antibody shot (1)
- ◯ Probably would not have my child get the antibody shot (2)
- ◯ Not sure about having my child get the antibody shot (3)
- ◯ Probably would have my child get the antibody shot (4)
- ◯ Definitely would have my child get the antibody shot (5)

Display This Choice:

If Q19 != 7

- ◯ I got the RSV vaccine during pregnancy (0)

Display This Question:

If Q22 = 1

Or Q22 = 2

Or Q22 = 3

© 2026 Porter RM et al. *JAMA Network Open*.

Q23 Which of the following, if any, are reasons that you ${Q22/ChoiceGroup/SelectedChoices}?
Please select all that apply:

- ▢ My child would not be at risk of getting RSV after being born (1)
- ▢ My child would not get very sick if they got RSV after being born (2)
- ▢ Worry about the short-term safety of an RSV antibody shot for my child (3)
- ▢ Worry about the long-term safety of an RSV antibody shot for my child (4)
- ▢ Worry that an RSV antibody shot might cause RSV (5)
- ▢ Worry that an RSV antibody shot might make future RSV infection worse (6)
- ▢ Worry about the cost of an RSV antibody shot (7)
- ▢ I've had RSV infection during this pregnancy (8)
- ▢ An RSV antibody shot would not work very well at preventing RSV for my child (9)
- ▢ Putting existing antibodies into someone is against my religious beliefs (10)
- ▢ I do not like needles (11)
- ▢ I do not think RSV is real (12)
- ▢ I wouldn't trust an RSV antibody shot (13)
- ▢ I wouldn't have time to have my child get the antibody shot (14)
- ▢ I feel like I don't know enough about RSV or the antibody shot to make a decision (15)
- ▢ My child's healthcare provider recommended against the antibody shot (16)

© 2026 Porter RM et al. *JAMA Network Open*.

☐ Other - please specify: (17) ____

Display This Question:

If Q22 = 1

Or Q22 = 2

Or Q22 = 3

Q24 If a healthcare provider strongly recommended your child get an RSV antibody shot, how likely would you be to have your child get the antibody shot after birth?

- ○ Definitely would not have my child get the antibody shot (1)
- ○ Probably would not have my child get the antibody shot (2)
- ○ Not sure about having my child get the antibody shot (3)
- ○ Probably would have my child get the antibody shot (4)
- ○ Definitely would have my child get the antibody shot (5)

Q25 If your child is born during the RSV season (in most places, October through March), your child may be able to get the RSV shot in the hospital or birthing center right after birth, or outpatient pediatrician's office within 1 week. Which option would you prefer?

- ○ Hospital or birthing center (1)
- ○ Outpatient pediatrician's office (2)
- ○ No preference (3)
- ○ Neither option (4)
- ○ Not sure (5)

© 2026 Porter RM et al. *JAMA Network Open*.

Q26 Hepatitis B vaccine is also recommended right after birth. Would you be willing to have your child vaccinated against both hepatitis B and get the RSV shot right after birth in the hospital or birthing center, if your healthcare provider recommends it?

- Both hepatitis B and RSV shot (1)
- Hepatitis B shot only (2)
- RSV shot only (3)
- Neither shot (4)
- Not sure (5)
- No preference (6)

Q27 If you could protect your child by getting the RSV vaccine during pregnancy and/or by giving your child an RSV antibody shot, which option would you choose?

- Vaccination during pregnancy (1)
- Antibody shot for child (2)
- No preference (3)
- Neither option (4)
- Not sure (5)

End of Block: RSV vaccination for child

© 2026 Porter RM et al. *JAMA Network Open*.

eTable 1: Distribution of reasons for negative/unsure intention to receive Respiratory Syncytial Virus (RSV) vaccine during pregnancy, among pregnant women.

	Pregnant Woman (n=68)	
	N[a]	col%[b]
**Reasons that you [definely would not/probably would not/not sure] get RSV vaccine during pregnancy**		
I feel like I don't know enough about RSV or the vaccine to make a decision	32	50.4
Worry about the safety of an RSV vaccine for my child	24	38.6
Worry about the safety of an RSV vaccine for myself	20	33.0
Worry that an RSV antibody shot might cause RSV	8	16.6
I wouldn't trust an RSV vaccine	10	13.4
My child would not be at risk of getting RSV after being born	9	11.6
I do not like needles	7	9.8
Worry that an RSV antibody shot might make future RSV infection worse	n<5	7.8
My child would not get very sick if they got RSV after being born	n<5	7.5
I am planning to get the RSV antibody shot for my child after they are born	n<5	2.9
Worry about the cost of an RSV vaccine	n<5	2.9
An RSV vaccine during pregnancy would not work very well at preventing RSV for my child after they are born	n<5	2.9
I've already had RSV	n<5	2.6
Vaccines are against my religious beliefs	n<5	2.2
I do not think RSV is real	n<5	1.4
My healthcare provider recommended against the RSV vaccine	n<5	1.2
I have troule finding a place that offers the RSV vaccine	n<5	0.9
Other	7	11.3

[a]Unweighted values represent actual numbers reported in dataset

[b]Weighted percentages calculated using geodemographic weights obtained from the panel provider

RSV = Respiratory Syncytial Virus

Cell size n < 5 have been supressed to preseve participant anonymity

© 2026 Porter RM et al. *JAMA Network Open*.

eTable 2: Distribution of reasons for negative/unsure intention to accept Respiratory Syncytial Virus (RSV) antibody shot for child, among pregnant women and parents.

	Pregnant		Parent of	
	N[a]	col%[b]	N[a]	col%[b]
**Reasons that you [definely would not/probably would not/not sure] get RSV antibody shot for child**				
I feel like I don't know enough about RSV or the antibody shot to make a decision	38	53.9	304	38.6
Worry about the long-term safety of an RSV antibody shot for my child	29	41.4	321	40.0
Worry about the short-term safety of an RSV antibody shot for my child	21	23.8	220	27.6
I wouldn't trust an RSV antibody shot	16	19.6	157	20.5
Worry that an RSV antibody shot might make future RSV infection worse	8	13.1	92	12.8
Worry that an RSV antibody shot might cause RSV	9	10.6	104	12.9
My child would not be at risk of getting RSV	10	9.5	52	8.0
I do not like needles	5	3.7	30	4.7
An RSV antibody shot would not work very well at preventing RSV for my child	n<5	3.1	42	5.6
My child's healthcare provider recommended against the antibody shot	n<5	2.2	21	5.1
Putting existing antibodies into someone is against my religious beliefs	n<5	2.2	21	1.9
I do not think RSV is real	n<5	1.1	11	1.2
I wouldn't have time to have my child get the antibody shot	n<5	1.0	9	1.7
My child would not get very sick if they got RSV	n<5	0.9	63	8.1
I've had RSV infection during this pregnancy	n<5	0.8	6	0.7
Worry about the cost of an RSV antibody shot	n<5	0.4	21	3.2
Other	5	7.3	74	10

[a]Unweighted values represent actual numbers reported in dataset

[b]Weighted percentages calculated using geodemographic weights obtained from the panel provider

RSV = Respiratory Syncytial Virus

Cell size n < 5 have been supressed to preseve participant anonymity

© 2026 Porter RM et al. *JAMA Network Open*.
