# Supplementary material for: Respiratory Syncytial Virus Immunization Intention During Pregnancy and Infancy
Source: JAMA Netw Open. 2026 Jul 24;9(7):e2625117. doi: 10.1001/jamanetworkopen.2026.25117 (PMC13401208; doi:10.1001/jamanetworkopen.2026.25117)
Supplement: Supplement 2. — Data Sharing Statement [file jamanetwopen-e2625117-s002.pdf]

Data Sharing Statement

Porter. Respiratory Syncytial Virus Immunization Intention During Pregnancy and Infancy. *JAMA Netw Open*. Published July 24, 2026. doi:10.1001/jamanetworkopen.2026.25117

Data
Data available: Yes
Data types: Deidentified participant data, Data dictionary
How to access data: lavanya.vasudevan@emory.edu
When available: With publication

Supporting Documents
Document types: None

Additional Information
Who can access the data: researchers whose proposed use of the data has been approved
Types of analyses: for any purpose
Mechanisms of data availability: with a signed data access agreement
